# Deep learning for epileptogenic zone delineation from the invasive EEG: challenges and lookouts

**DOI:** 10.1093/braincomms/fcab307

**Published:** 2021-12-27

**Authors:** Sem Hoogteijling, Maeike Zijlmans

**Affiliations:** Department of Neurology and Neurosurgery, Brain Center, University Medical Center Utrecht, Utrecht, The Netherlands; Stichting Epilepsie Instellingen Nederland (SEIN), Heemstede, The Netherlands; Technical Medicine, University of Twente, Enschede, The Netherlands; Department of Neurology and Neurosurgery, Brain Center, University Medical Center Utrecht, Utrecht, The Netherlands; Stichting Epilepsie Instellingen Nederland (SEIN), Heemstede, The Netherlands

## Abstract

This scientific commentary refers to ‘Refining epileptogenic high-frequency oscillations using deep learning: a reverse engineering approach’ by Zhang *et al*. (https://doi.org/10.1093/braincomms/fcab267).

**This scientific commentary refers to ‘Refining epileptogenic high-frequency oscillations using deep learning: a reverse engineering approach’ by Zhang *et al*. (https://doi.org/10.1093/braincomms/fcab267)**.

Deep learning (DL) has shown increasingly high-level performances in the medical domain, especially in the field of medical imaging.^1^ In the past decade, DL has also found its way in the automatic evaluation of the EEG, for example, in epilepsy for the detection of seizures.^2^ Recently, researchers attempted DL approaches for delineation of the epileptogenic zone (EZ) from the invasive EEG (iEEG).^2,3^ This is relevant, as manual EZ delineation remains challenging, because the clinician needs to process and interpret complex and extensive information including information that may be present but hidden to the human eye.^4^ A DL approach for EZ delineation comes with several challenges. We will discuss challenges encountered in the study from Zhang *et al*.^5^ published recently in *Brain Communications*. We start off with a brief overview of artificial intelligence (AI) in medical healthcare.

AI encompasses all methods that aim to mimic human cognitive functioning, e.g. make a diagnosis from computed tomography images. Machine learning (ML) is an overarching term within AI that refers to methods that learn through experience using data. There are three types of learning in ML: supervised learning, unsupervised learning and reinforcement learning (Fig. 1). With supervised learning, the data are labelled, and an ML model is trained to predict these labels, e.g. a diagnosis or life expectancy. In unsupervised learning, the data are not labelled, and the ML model is trained to find hidden structures in the data and discover clusters of subgroups. In reinforcement learning, an ML model learns the optimal behaviour in a certain environment, e.g. automated robotic surgery. For each type of learning, multiple ML models exist and the most suitable model to use depends on the task at hand. For some ML models, features need to be extracted from the raw data, which requires expert knowledge about the data and the ML problem. In DL, a subfield of ML, successive layers are used to transform the input data into more meaningful representations, where the first layers of a DL model act as feature extractors. Hence, DL models do not require any feature engineering. This DL property makes a DL approach interesting for EZ delineation, given that no single EZ iEEG feature or combination of features yields enough predictive information for clinical use.^6^

**Figure 1:**
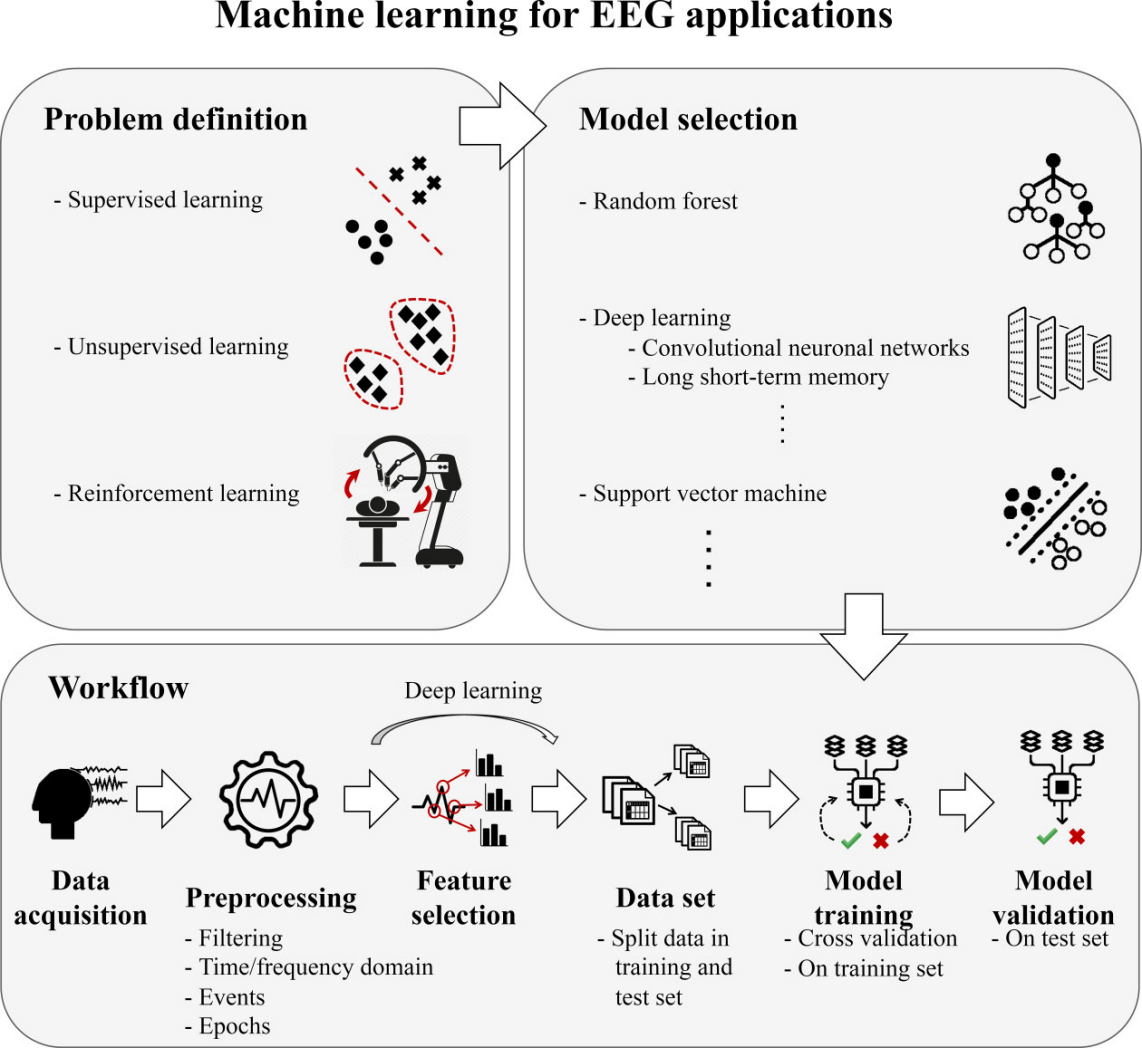
**An overview of ML approaches**. First the ML problem is defined and an appropriate ML model is chosen. Subsequently, the data are preprocessed, e.g. filter the data, transformation to the frequency domain and selecting events or epochs. Next, features are selected or deep learning is used in which the raw data is used. The data are then split into a training and test set. The model is trained based on the training data using CV. Finally, the performance of the model is assessed on unseen data, the test set. This figure illustrates the choices made during an ML approach, such as model selection, preprocessing steps, feature selection, percentage split in CV and training of the model (which also includes optimizing hyperparameters of the model).

Several DL approaches for EZ delineation have been proposed. San-Segundo *et al*.^2^ and Daoud and Bayoumi^3^ used a DL approach to classify whether iEEG epochs from the Bern-Barcelona data set^7^ and the Bonn data set^8^ were part of the EZ. Both obtained excellent results with accuracies above 90%. The generalizability of these studies can be questioned as these data sets consist of iEEG recordings from five temporal lobe epilepsy patients. Other DL approaches have been proposed for automatic detection of EZ biomarkers such as interictal epileptiform discharges^9^ and high-frequency oscillations (HFOs).^10^

Zhang *et al*.^5^ used a DL approach to classify dichotomously whether an HFO was located on a spike (spike HFO) because spike HFOs are regarded more epileptogenic than non-spike HFOs. They used two types of validation methods to assess the DL model’s generalizability, namely patient-wise cross-validation (CV) and all-patient CV. In their patient-wise CV, the model was trained on HFOs from a subset of patients and validated on the HFOs of the remaining patients. In all patients CV, the HFOs from all patients were pooled together, and the model was trained on a subset of these pooled HFOs and validated on the remaining pooled HFOs. This latter type of CV was also seen in the studies of San-Segundo *et al*.^2^ and Daoud and Bayoumi,^3^ probably due to the meagre number of patients to construct a training and validation set with enough patients and the laborious work of annotating the iEEG. However, since HFOs within one patient are similar, validating a DL model on data from the same patients whose data were used in training will affect the reliability of the generalization assessment to other patients. This is substantiated by the reported performance difference between patient-wise CV and all patients CV for classification of a spike HFO in the study of Zhang *et al*.:^5^ the mean (standard deviation) of the precision: 81.4% (16.1%) versus 92.0% (0.8%). Patient-wise CV is hence the most adequate assessment of a DL model’s generalizability for clinical use in different patients.

The automatic detection of HFOs in the iEEG addresses the tedious work of manually marking these biomarkers. Nonetheless, HFOs are also generated by healthy tissue, which may limit their specificity as EZ biomarker.^11^ This have led Zhang *et al*.^5^ to use DL to refine epileptic HFOs, in which HFOs from resected tissue were labelled as epileptic and HFOs from preserved channels as non-epileptic in nine patients who underwent epilepsy surgery with postsurgical seizure freedom. They did not report the classification results but performed multiple group level analyses based on these identified ‘epileptic’ HFOs. As these analyses were on a group level, it is unknown if these identified ‘epileptic’ HFOs can classify an individual iEEG epoch as part of the EZ and can hence aid in EZ delineation. It would have been interesting to know if the DL model could discriminate between HFOs originating from the EZ and from healthy tissue. Especially since much research on HFOs consists of analysis on the group level and not individual level. Hence, the true clinical value of HFOs and the ‘epileptic’ HFOs found by Zhang *et al*.^5^ for an individual patient remains pending.

All previous DL studies for EZ delineation have focused on classifying a single channel or a biomarker from a single channel as part of the EZ or not. One should not forget that the first assumption that is made when choosing an ML or DL approach is that the information for the task at hand is present in the data that are given to the DL model. Since epilepsy is regarded as a network disorder, classifying a single iEEG channel as part of the EZ without taking adjacent channels in consideration may be impossible. Providing neighbouring channels to the DL model may improve EZ delineation performances. Furthermore, the approach of interpreting the resected area as the EZ may lead to labelling errors, given that the resected area may be larger than the actual EZ. More complex learning methods (e.g. multi-instance learning, which is a generalization of traditional supervised learning) may address these labelling errors.

Once a DL model for EZ delineation is successfully trained, assessing the features learnt by the DL model can be interesting for clinical implementation and research purposes. Zhang *et al*.^5^ tried to identify features from the found ‘epileptic’ HFOs based on prior knowledge. They found that epileptic HFOs were often accompanied by a spike and showed an inverted T-shape in the time–frequency plots. However, around 30% of non-epileptic HFOs also had spikes and adding the inverted T-shaped feature to non-epileptic HFO did increase the model’s prediction towards an epileptic HFOs by just 0.285 on average. Observing these features is therefore not enough to classify an individual HFO as an epileptic HFO. More sophisticated methods that can explain a DL model which do not require prior assumptions may reveal novel EZ iEEG features in the future. Explainable AI (xAI) refers to such methods that aim to provide an explanation along with the output of an ML model.^12^ These methods can provide a saliency map that highlights important aspects in the input according to the DL model. Novel EZ biomarkers in the iEEG may be discovered by using xAI and a DL model which classifies iEEG signals as part of the EZ. In addition, the saliency maps can be a good interpretation tool for neurologists, which they can use to observe the reasons why a DL model is given a certain recommendation.

In summary, DL for EZ delineation from the iEEG is a promising approach as no *a priori* feature extraction is required and it is an individual-level analysis. The required large iEEG data for DL may be difficult to obtain, since well-documented iEEG data are scarce, and labelling the data for resected areas and artefacts is laborious. Nonetheless, a proper data set is essential for a DL model to learn generalizable patterns and to get a reliable assessment of a DL model’s generalizability. Therefore, more publicly available iEEG data sets are of great value. With enough patients, it can be assessed whether DL can be used to classify a single iEEG channel as part of the EZ or incorporation of more channels is needed. When a DL model is successfully trained, xAI may reveal previously unknown EZ features and may aid in the DL model’s clinical implementation. Further down the line, a DL model and xAI may guide the surgeon in the operation theatre to resect only epileptic tissue and improve postsurgical seizure freedom results.

## Competing interests

The authors report no competing interests.

## Funding

MZ is funded by ERC starting grant 803880.
